# Managing Fatigue: Experiences From a 6-week Course for Adults With Cerebral Palsy

**DOI:** 10.1016/j.arrct.2023.100300

**Published:** 2023-10-02

**Authors:** Ellinor Nilsson, Séverine Hedberg Dubuc, Nazdar Ghafouri, Anne Söderlund Schaller

**Affiliations:** a Department of Habilitation, and Department of Biomedical and Clinical Sciences, Linköping University, Motala, Sweden; b Pain and Rehabilitation Centre, and Department of Health, Medicine and Caring Sciences, Linköping University, Linköping, Sweden; c Division of Nursing Science, Department of Health, Medicine and Caring Sciences, Linköping University, Linköping, Sweden

**Keywords:** Cerebral palsy, Fatigue, Patient education, Rehabilitation, Self-management

## Abstract

**Objective:**

To explore experiences of a 6-week Fatigue Management course (FMC) in adults with cerebral palsy (CP).

**Design:**

A qualitative study using semi-structured interviews. The study process followed the Consolidated Criteria for Reporting Qualitative Research (COREQ).

**Setting:**

The study was conducted in southeastern Sweden in an out-patient setting.

**Participants:**

Adults (N=8) with CP who had participated in FMC.

**Interventions:**

Not applicable.

**Main Outcome Measure:**

Qualitative content analysis of the transcribed interviews led to identification of a main category, categories, and subcategories, describing the participants’ experiences of FMC.

**Results:**

The analysis identified 2 categories: Awareness regarding fatigue, with the 2 subcategories: A better understanding, and The feeling of not being alone; and Perceive opportunities for changes, with the 3 subcategories: Understanding the need for changes, Demanding process, and Taking steps toward change. These categories were summed up in the main category describing the participants’ experiences of FMC: A challenging and eye-opening course that gave deeper self-understanding and thoughts about making changes.

**Conclusions:**

Overall, the participants described positive experiences of FMC, with increased awareness regarding fatigue and insight regarding the possibilities for change. Nevertheless, there were challenges in coping with the extensive information and with the home assignments. This study gives promising results regarding the applicability of FMC for adults with CP. However, there is a need for course modifications with more targeted and differentiated content that is manageable and does not overload the participants. The modifications should include extended time, the addition of individual support, and follow-up between sessions, to increase participants’ opportunities to implement new strategies and initiate behavioral change.

Fatigue is a complex symptom that describes a “reduced capacity to sustain force or power output (physiological), reduced capacity to perform multiple tasks over time (psychological), and simply a subjective experience of feeling exhausted, tired, weak or having lack of energy”.^1^ In adults with cerebral palsy (CP), fatigue is a significant health issue, associated with perceived poor health.^2^^,^^3^ Up to 40% experience fatigue^4^, ^5^, ^6^ and report more severe fatigue than the general population.^4^^,^^5^^,^^7^ The severity of fatigue seems to be greater in those with greater functional impairment according to the Gross Motor Function Classification System (GMFCS).^8^^,^^9^ The GMFCS is spread over 5 levels (I-V), where higher levels indicate more limitations.^10^ Studies also indicate that subtype of CP is a determinant with more severe fatigue in those with spastic bilateral CP compared with unilateral.^6^^,^^11^ Individuals with CP experience both physical and/or psychological fatigue, often co-occurring with pain and depressive symptoms, making it a complex and multifaceted experience.^5^ The most common factors contributing to CP-related fatigue are activity-related factors, general demands of life, and poor sleep/rest.^12^ While fatigue is a prevalent concern in this population, the understanding of the mechanisms underlying fatigue remains limited,^7^ making it challenging to accurately estimate and assess fatigue in individuals with CP.^13^

Few studies investigate treatments of fatigue for this population. However, some studies have found that physical activity-based interventions can help mitigate fatigue.^14^^,^^15^ This finding is consistent with literature regarding the treatment of other disease-related fatigue such as multiple sclerosis (MS) and cancer.^16^, ^17^, ^18^ There is a need to set up effective intervention programs and education in self-manage fatigue and target factors contributing to CP-related fatigue.^3^^,^^7^^,^^12^

Education in self-management can be effective on fatigue and improve quality of life for patients with MS and cancer.^19^ The course *Managing Fatigue: A Six-week Course for Energy Conservation*^20^ has been found to help patients with MS manage with fatigue more effectively.^21^, ^22^, ^23^ The manual has been translated into Swedish.^24^ The purpose of this course is to provide knowledge about fatigue and increase ability to self-manage everyday activities. It focuses on the importance of rest and the use of strategies, techniques, tools, and body mechanics to reach activity balance. The course spans over 6 weeks, with one 2-hour session per week. Between sessions, participants are assigned homework related to the topics covered in the different sessions. The content of the course is modifiable and can be adjusted depending on the participants’ needs. To our knowledge, self-managing education, including *Managing Fatigue*, has not been evaluated for CP-related fatigue. This is essential to gain a better understanding of how education could be structured and which parts individuals with CP consider relevant and helpful.

This qualitative interview study aimed to explore experiences of the Swedish version of the Fatigue Management course (FMC) in adults with CP-related fatigue.

## Methods

The consolidated criteria for reporting qualitative research^25^ statement was used to guide this qualitative inductive interview study. The Swedish Medical Ethical Board approved the study (Dnr 2019-06510) and all participants gave written, informed consent.

### Participants and setting

The participants were selected using a convenience sample. Inclusion criteria were ≥18 years of age, diagnosis of CP, and having a current contact with a habilitation center in Sweden. Exclusion criteria was intellectual disability. Of the 40 people identified and invited by mail, 21 came to an individual pre-meeting, where additional information about FMC and the study was given as well as assessment of fatigue using Fatigue Severity Scale (FSS).^26^^,^^27^ This 9-item assessment is designed to measure some aspects of both physical and cognitive fatigue experienced over the past week. Respondents rate their experiences on a 7-point Likert scale, where higher score indicate greater fatigue.^13^ FSS has not been evaluated for persons with CP, but for other health conditions.^13^^,^^26^^,^^27^ It is also widely used in Sweden for persons with CP, being a part of the National Quality Register for CP.^28^ According to the FMC-manual,^24^ a person with a mean of ≥4 (min 1, max 7) on the FSS could be included in FMC. Ten persons with a mean of ≥4, and 1 person with a mean of 3.78, were asked to participate in the study. Of these, 3 declined, and 8 agreed and were included in the study. The participants attended FMC lead by an occupational therapist and psychologist, and in one session, a physical therapist. The course was to some extent modified, in order meet the needs of individuals with CP. The first session contained information about CP-related fatigue, and the second focused on body mechanics based on CP, relaxation, and body awareness. Except for these adjustments, the course followed the FMC-manual.^24^

Table 1 describes the participants 6 weeks before FMC. The median age was 30 years and 50% were women. Five had spastic unilateral CP and 3 bilateral with GMFCS levels I-II. Four of the participants had paid work, one studied, and three worked with wage subsidy. The median total score on the FSS was 4.9 (3.78-6.22).

**Table 1:** Characteristics of the participants

	Total (N=8)
Sex (female/male), n	4/4
Median age, years (range)	30 (24-57)
Cohabiting/single, n	3/5
Education, median years (range)	12 (9-15)
Paid work/Wage subsidy/Workplace training/Student, n	4/2/1/1
Housing support, n	2
Type of CP; SUCP/SBCP	5/3
GMFCS-level; I/II	5/3
Other diseases; Mental illness/Epilepsy	4/3
FSS, median total score (range)	4.9 (3.78-6.22)
*Fatigue causes frequent problems for me*, median (range)	5.5 (4-7)
*Fatigue is among my 3 most disabling symptoms*, median (range)	6 (4-7)

Abbreviations: SBCP, spastic bilateral cerebral palsy; SUCP, spastic unilateral cerebral palsy.

### Interviews

The present study used a semi-structured interview guide based on Kvale and Brinkmann,^29^ with questions focused on the participants’ experiences of participating in FMC. The opening question was “Can you describe your experiences participating in FMC?”. Additional questions were asked to cover all relevant themes. A pilot interview was conducted, leading to the addition of two questions. This interview had good quality and relevance and was included. The interviews were conducted 1-2 weeks after FMC by the first author either at the habilitation center (n=7) or in the participant's home (n=1), and lasted for 17-52 minutes (median 36 minutes). There was no one else present during the interviews. The interviews were audio-recorded and transcribed by the first author.

### Data analysis

The transcribed interviews were analyzed by the first (E.N.) and last author (A.S.S.), using qualitative content analysis.^30^ To gain a sense of the whole, the interviews were read systematically several times with an inductive approach to identify meaning units of the text corresponding to the aim of the study. Notes were written in the text as part of the open coding process and the meaning units were compared and discussed until agreement was achieved. Codes were created from the meaning units, then into subcategories and categories, and later summed into a main category. All authors discussed the categories to ensure their consistency and correspondence to the aim of the study until consensus was reached.

## Results

**Fig 1:**
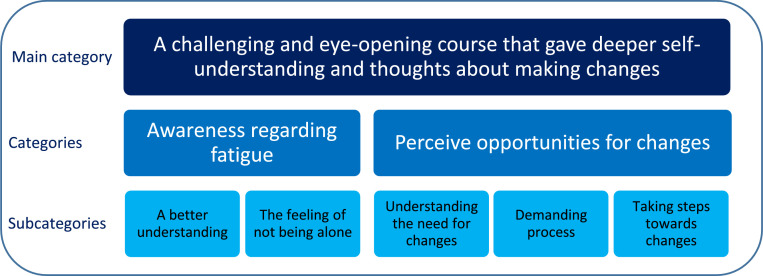
The result of this study showing the participants’ experiences from the Fatigue Management course, divided into a main category, categories and subcategories.

The analysis of the interviews resulted in the identification of a main category, two categories, and five subcategories describing the participants’ experiences of FMC (fig 1). Overall, the participants experienced FMC as a challenging and eye-opening course. They described experiencing an enhanced awareness regarding fatigue, which resulted in a deeper self-understanding and the feeling of not being alone. Attending the course stimulated contemplation about making changes. However, participants expressed that carrying out the implementation was challenging because of managing the extensive information presented in FMC, along with the demanding home assignments.

In this section, each of these categories are described in depth and supported with quotations from the interviews. The participant's number (P1 for participant 1, and so on) is presented with each quotation.

### Awareness regarding fatigue

#### A better understanding

Many of the participants described that FMC gave new knowledge regarding fatigue and that it was a positive experience to receive this information. There was an overall appreciation to get a deeper understanding about the problems experienced. Most described a feeling of recognition and an “aha moment”.

“It was in the beginning, you got an aha-experience first meeting when you were told like this is when you have fatigue [. . .] I could recognize myself so much in it” (P7).

Some of the participants expressed a wish of additional medical information when it comes to CP-related fatigue to gain an even deeper understanding. However, one described it as depressing and hard to accept.

“You focused a lot on the problem, I understand that you have to, but I almost get worse from focusing on the problem, I thought it was a bit scary” (P1).

#### The feeling of not being alone

Sharing experiences with others in similar situations was positively described by many with a feeling of easiness of understanding others’ perspective. Some found that hearing others describe how fatigue affected functioning and daily living helped them reach a deeper understanding of their own fatigue. Moreover, this provided them with a sense of companionship, and most participants found this interaction to be the most fulfilling aspect of FMC.

“It was nice to get confirmation from others that I am not alone in this” (P4).

“It wasn't always that the course leader had the right answer, it turned out that you got the answer from one of the participants and then a discussion was opened” (P3).

However, some felt that the time for one-to-one discussions was too short for a deeper conversation and exchange of experiences.

### Perceive opportunities for changes

#### Understanding the need for changes

Most described that FMC gave an insight regarding a need for changes. Some found that their new understanding and acceptance contributed to that.

“I have understood that it is not possible to work full time anymore. I had a lot of resistance to it, strange, but now I feel, well okay” (P8).

Some described a new mindset about the challenges associated with fatigue, such as being more aware and accepting of what their body was communicating. The course helped them understand their own needs and insights regarding activity balance.

“It's that I stop and maybe need more rest–in other words, I listen more to my body than I did before; the recovery is more important” (P7).

“You do not have to work so hard or raise the gear level more, I start to get a little more insight into that and that is what I take with me” (P6).

A few of the participants described that despite FMC provided an insight into the need for changes, difficulties remained in knowing where to start and the feeling of not having the opportunity to change one's life situation. In addition, FMC gave increased awareness of the importance to communicate one's challenges with family, friends, and other people. This was seen as difficult, and some wanted more support in how to communicate fatigue.

“It is important to communicate with people in the surroundings what you feel, that you need rest, to set limits” (P2).

#### Demanding process

About half of the participants found the amount of information and work in the course too much to cope with during the allotted time. Some stated that this made it hard to follow.

“I thought it was good, but at the same time I felt I was way too tired” (P8).

The length of each meeting was manageable although a few felt it was too long, leaving them with a high level of fatigue and difficulties following the discussions. Some participants found that FMC had too many parts and too much training, while others’ felt that the course's pace and content was good.

“Maybe it would have worked with one and a half hour max; otherwise, I feel it's a long time [. . .] I lose concentration so it's not sure that I hear everything [. . .] I see the person talking, but then nothing goes in, it doesn't” (P1).

“It was not too much not too little information, but on the other hand I sometimes have a hard time to find strength when there is too much new information, but for my part, personally, I feel it was just enough” (P2).

A majority felt that the homework was challenging. The instructions were hard to understand and some did not seem relevant.

“Some of the homework was a bit hard to understand, what we were supposed to do, and I think that the other participants also felt that [. . .] most of us looked like question marks when we left. I read the instructions several times at home but couldn't understand what to do” (P5)*.*

A few stated that having homework increased their fatigue and felt like yet one more thing that needed to be done. Participant 5 mentioned that the homework was sometimes based on previous insight, so if one did not already have some strategies, the homework was difficult to complete. However, others did not experience difficulties understanding the homework and found possibilities to customize it. Most agreed that homework was important, but wanted more individualized instructions and opportunity to work on them during the sessions.

#### Taking steps toward changes

Most participants felt that FMC made them aware of ways to manage fatigue better that they found useful. Making schedules to help prioritize, sorting activities, and finding time for recovery/rest, were frequently mentioned as valuable. Some described that they would start using these and some had already started. However, one participant expressed some anxiety about the thought of making changes.

“The thought of resting, for me it was just ‘resting? What? Where shall I rest?’ at first. Because it's not how I've been living, it has just been all-in. So to find space for rest was the first thing” (P6).

A schedule increased concentration and was mentioned as helpful for becoming more aware of how to plan activities and rest in a more balanced way. Becoming more aware about how activities strain physically and mentally in different ways and require different recovery was mentioned as helpful. Some listed that dividing activities into smaller parts spread over more than one day simplified the activities.

“I've learned that I need to slow down [. . .] and when it comes to some chores at home, I might have to split them up on more than one day. Maybe I can't clean all rooms, do the laundry and the dishes on the same day” (P3).

On the contrary, some described that the proposed tools from FMC were challenging to implement and could not fit into their everyday life, creating a feeling of hopelessness.

About half of the participants stated a need for support to continue the work of managing fatigue. FMC provided a good basis for information and a better understanding; however, individual support was requested to move forward.

“In this course you got to know it, then you have a little idea of what you might need to look into, but well much like after surgery you set up a rehabilitation plan how to get back, that you continue from having the basics to address my specific problem” (P5).

## Discussion

Overall, the participants had positive experiences with FMC. Most gained a better understanding of their own situation, and expressed a feeling of recognition when learning about fatigue. Although fatigue is a recognized and prevalent health issue in adults with CP,^2^, ^3^, ^4^, ^5^, ^6^, ^7^, ^8^, ^9^ the participants believed that the correlation between CP and fatigue had not been previously addressed for them. While this result cannot be generalized given its small sample, it is still an interesting observation to highlight, indicating a greater need for information to patients with CP. Furthermore, most of the participants expressed that meeting others in similar situation contributed to the feeling of not being alone and was the most rewarding thing about FMC. Participants shared experiences and tools with each other, which is believed to be one of the factors contributing to perceiving opportunities for changes. The benefit of peer interaction (ie, meeting and sharing experiences with others) when addressing issues of fatigue aligns with earlier research outcomes.^19^^,^^21^^,^^31^ Providing useful information, FMC increased insight which helped most participants accept fatigue. This made them think and relate to their symptoms in a new way that supported taking steps toward changes. Some said that they received tools and strategies to manage fatigue to use further on. In line with Mathiowetz et al,^21^ making schedules for the day and make time for rest were mentioned as effective strategies. Nonetheless, some participants wanted more individual support to help them move forward and address their specific problems and solutions. In addition, a majority felt that the homework was difficult, both regarding understanding how to do it and the feeling that it was not relevant. We highlight the possibility that the participants would benefit from the homework more if it was performed during the end of each session with support from the course leaders rather than at home. That is, instructions could be clarified and the task more person-centered. Individual support between each session could have further helped the participants meet the challenges associated with implementing new strategies. The advantage of adding individual support and giving time for homework during sessions has been highlighted by Van Heest et al.^31^ There is also a need of understanding the mechanisms behind fatigue in individuals with CP to better address their specific needs.^13^

Overall, FMC was demanding, and some felt that the amount of information was too much to cope with. With this follows the risk of not being able to take advantage of the new knowledge as well as increased fatigue. The cognitive functioning is often affected in individuals with CP, even if the extent and severity of these impairments vary significantly, spanning from severe and global, that is, intellectual disability, to limited challenges in a specific domain.^32^^,^^33^ Considering these challenges existing for individuals with CP, together with the participants’ experiences, we highlight the need to modify FMC. For example, the duration of each session could be reduced but with the addition of extra sessions, leaving each session with less information. The population of individuals with CP is highly heterogeneous,^34^ thus the approach in FMC could also be more targeted and differentiated to address the unique needs of each individual, which would facilitate their process of making changes, as well as avoid overloading the participants with unnecessary information. The duration and dose of self-managing education regarding fatigue has been discussed recently as an important part in receiving larger effects, especially because it aims for behavioral changes.^19^^,^^35^

We did not find previous studies evaluating fatigue self-management education targeting CP-related fatigue. The strengths of this study include patient-reported data and a study design that reflects real-world experiences. We believe that there is an added value in gaining better insight into how adults with CP-related fatigue experience participation in FMC as this knowledge could be used to optimize the educational experience.

### Study limitations

The methodology of our interview study was well suited for capturing participants’ experiences and thoughts about FMC.^36^ There are, however, some limitations. First, this study was based on a relatively small sample. Although the population, adults with CP and fatigue, is limited, the interviews had extensive content ensuring adequate data. The GMFCS-level of the participants was low (I-II), and studies indicate that individuals with higher GMFCS-levels also experience more fatigue.^8^^,^^9^ In this study, intellectual disability was an exclusion criteria, which may have contributed to the limitation of a broader sample regarding GMFCS-level. Second, the results from qualitative studies cannot usually be generalized. However, the purpose of this study was not to generalize but to explore the experiences of a specific course. All interviews were semi-structured and conducted with the help of an interview guide to ensure that important areas were discussed. The same question was used to begin each interview. Confirmatory questions were asked during the interviews, which is a way of clarifying and correcting potential misunderstandings.^37^ All interviews were performed by the first author (E.N.), which is a strength of the study as it facilitated a similar approach in the interview situation. This author also transcribed all the interviews and therefore was familiar with the data. Third, preunderstanding on the part of the first author was considered. The author's knowledge of the topic and personal knowledge, as a physiotherapist, of a few of the participants may have affected the research process. However, it can also be seen as an advantage as such knowledge and experience can contribute to relevant confirmatory questions. Also, several researchers evaluated the analysis to ensure other views of the data were considered. These researchers were from different areas such as physiotherapy, nursing, medicine, and psychology. This could be seen as “investigator triangulation” and therefore a strength.^38^

## Conclusions

This study described FMC as a way of increasing awareness and insights regarding fatigue as well as a way to develop strategies to manage fatigue for individuals with CP. The results provide a basis for further development of self-management education programs. However, it is clear that modifications are needed to optimize the effectiveness of these programs. These modifications should offer a targeted, individualized information that aligns with the needs of individuals with CP. Additionally, it is important to extend the duration of the program, introduce individual support, and add follow-up between sessions. These changes will not only empower participants but also prevent information overload, paving the path to implementation of new strategies and behaviors. Further research, contributing with advanced comprehension of fatigue in individuals with CP, is essential for the development of a more tailored course. However, this study gives promising results regarding the applicability of FMC in the health care of CP-related fatigue.

## References

[1] Kaasa S, Loge JH, Knobel H, Jordhøy MS, Brenne E (1999). Fatigue. Measures and relation to pain. Acta Anaesthesiol Scand.

[2] Benner JL, Hilberink SR, Veenis T, Stam HJ, van der Slot WM, Roebroeck ME (2017). Long-term deterioration of perceived health and functioning in adults with cerebral palsy. Arch Phys Med Rehabil.

[3] van Gorp M, Hilberink SR, Noten S (2020). Epidemiology of cerebral palsy in adulthood: a systematic review and meta-analysis of the most frequently studied outcomes. Arch Phys Med Rehabil.

[4] Jahnsen R, Villien L, Stanghelle JK, Holm I (2003). Fatigue in adults with cerebral palsy in Norway compared with the general population. Dev Med Child Neurol.

[5] Van Der Slot WM, Nieuwenhuijsen C, Van Den Berg-Emons RJ (2012). Chronic pain, fatigue, and depressive symptoms in adults with spastic bilateral cerebral palsy. Dev Med Child Neurol.

[6] Russchen HA, Slaman J, Stam HJ, van Markus-Doornbosch F, van den Berg-Emons RJ, Roebroeck ME (2014). Focus on fatigue amongst young adults with spastic cerebral palsy. J Neuroeng Rehabil.

[7] Puce L, Pallecchi I, Chamari K (2021). Systematic review of fatigue in individuals with cerebral palsy. Front Hum Neurosci.

[8] Brunton LK (2018). Descriptive report of the impact of fatigue and current management strategies in cerebral palsy. Pediatr Phys Ther.

[9] McPhee PG, Brunton LK, Timmons BW, Bentley T, Gorter JW (2017). Fatigue and its relationship with physical activity, age, and body composition in adults with cerebral palsy. Dev Med Child Neurol.

[10] Palisano R, Rosenbaum P, Walter S, Russell D, Wood E, Galuppi B (1997). Development and reliability of a system to classify gross motor function in children with cerebral palsy. Dev Med Child Neurol.

[11] Lundh S, Nasic S, Riad J (2018). Fatigue, quality of life and walking ability in adults with cerebral palsy. Gait Posture.

[12] Brunton LK, McPhee PG, Gorter JW (2021). Self-reported factors contributing to fatigue and its management in adolescents and adults with cerebral palsy. Disabil Rehabil.

[13] Dutia I, Eres R, Sawyer SM (2023). Fatigue experienced by people with cerebral palsy: a systematic review of assessment tools and decision tree. Disabil Rehabil.

[14] Vogtle LK, Malone LA, Azuero A (2014). Outcomes of an exercise program for pain and fatigue management in adults with cerebral palsy. Disabil Rehabil.

[15] Slaman J, van den Berg-Emons HJ, van Meeteren J (2015). A lifestyle intervention improves fatigue, mental health and social support among adolescents and young adults with cerebral palsy: focus on mediating effects. Clin Rehabil.

[16] Tomlinson D, Diorio C, Beyene J, Sung L (2014). Effect of exercise on cancer-related fatigue: a meta-analysis. Am J Phys Med Rehabil.

[17] Oberoi S, Robinson PD, Cataudella D (2018). Physical activity reduces fatigue in patients with cancer and hematopoietic stem cell transplant recipients: a systematic review and meta-analysis of randomized trials. Crit Rev Oncol Hematol.

[18] Asano M, Finlayson ML (2014). Meta-analysis of three different types of fatigue management interventions for people with multiple sclerosis: exercise, education, and medication. Mult Scler Int.

[19] Hersche R, Roser K, Weise A, Michel G, Barbero M (2022). Fatigue self-management education in persons with disease-related fatigue: a comprehensive review of the effectiveness on fatigue and quality of life. Patient Educ Couns.

[20] Packer L, Brink N, Sauriol A (1995).

[21] Mathiowetz V, Matuska KM, Murphy ME (2001). Efficacy of an energy conservation course for persons with multiple sclerosis. Arch Phys Med Rehabil.

[22] Mathiowetz VG, Finlayson ML, Matuska KM, Chen HY, Luo P (2005). Randomized controlled trial of an energy conservation course for persons with multiple sclerosis. Mult Scler.

[23] Sauter C, Zebenholzer K, Hisakawa J, Zeitlhofer J, Vass K (2008). A longitudinal study on effects of a six-week course for energy conservation for multiple sclerosis patients. Mult Scler.

[24] Packer L, Brink N, Sauriol A (2017).

[25] Tong A, Sainsbury P, Craig J (2007). Consolidated criteria for reporting qualitative research (COREQ): a 32-item checklist for interviews and focus groups. Int J Qual Health Care.

[26] Krupp LB, LaRocca NG, Muir-Nash J, Steinberg AD (1989). The Fatigue Severity Scale. Application to patients with multiple sclerosis and systemic lupus erythematosus. Arch Neurol.

[27] Mattsson M, Möller B, Lundberg I, Gard G, Boström C (2008). Reliability and validity of the Fatigue Severity Scale in Swedish for patients with systemic lupus erythematosus. Scand J Rheumatol.

[28] CPUP: a follow-up surveillance programme for people with cerebral palsy (CP) 7 September 2023]. Available at: URL: https://cpup.se/in-english/what-is-cpup-in-english/. Accessed September 7, 2023.

[29] Kvale S (1997). Interviews : an introduction to qualitative research interviewing.

[30] Elo S, Kyngas H (2008). The qualitative content analysis process. J Adv Nurs.

[31] Van Heest KNL, Mogush AR, Mathiowetz VG (2017). Effects of a One-to-One Fatigue Management Course for people with chronic conditions and fatigue. Am J Occup Ther.

[32] Stadskleiv K (2020). Cognitive functioning in children with cerebral palsy. Dev Med Child Neurol.

[33] Straub K, Obrzut JE (2009). Effects of cerebral palsy on neuropsychological function. J Dev Phys Disabil.

[34] Bax M, Goldstein M, Rosenbaum P (2005). Proposed definition and classification of cerebral palsy, April 2005. Dev Med Child Neurol.

[35] Kim S, Xu Y, Dore K, Gewurtz R, Larivière N, Letts L (2022). Fatigue self-management led by occupational therapists and/or physiotherapists for chronic conditions: a systematic review and meta-analysis. Chronic Illn.

[36] Kvale S (1997).

[37] Kvale S (2008).

[38] D F Polit and C T Beck, *Nursing Research : Generating and Assessing Evidence for Nursing Practice.* Eleventh edition ed. Philadelphia, PA: Wolters Kluwer Philadelphia, PA, 2021.

